# Effect of antenatal care on low birth weight: a systematic review and meta-analysis in Africa, 2022

**DOI:** 10.3389/fpubh.2023.1158809

**Published:** 2023-06-27

**Authors:** Garedew Tadege Engdaw, Amensisa Hailu Tesfaye, Maru Feleke, Aragaw Negash, Amanuel Yeshiwas, Wabiw Addis, Dessie Abebaw Angaw, Melaku Tadege Engidaw

**Affiliations:** ^1^Department of Environmental and Occupational Health and Safety, Institute of Public Health, College of Medicine and Health Sciences, University of Gondar, Gondar, Ethiopia; ^2^Amhara Regional Health Beauro, Wogera Primary Hospital, Northwest, Ethiopia; ^3^Department of Epidemiology and Biostatistics, Institute of Public Health, College of Medicine and Health Sciences, University of Gondar, Gondar, Ethiopia; ^4^Department of Public Health, College of Health Sciences, Debre Tabor University, Debre Tabor, Ethiopia

**Keywords:** antenatal care, low birth weight, Africa, public health, institutional births

## Abstract

**Background:**

Risk identification, as well as the prevention and management of diseases associated with pregnancy or other conditions that may occur concurrently, is the essential component of ANC.

**Method:**

The observational follow-up and cross-sectional studies on the effect of antenatal care on low birth weight in Africa were conducted according to the Preferred Reporting Items for Systematic reviews and Meta-Analyses guidelines. Five computerized bibliographic databases: Google Scholar, PubMed, Scopus, Cochrane Library, and Hinari Direct were searched for published studies written in English till May 2022. The risk of bias assessment tools developed by the Joanna Briggs Institute for cross-sectional and observational follow-up research was used, and the caliber of each included study was assessed. Seven papers were included, with a total of 66,690 children participating in the study.

**Results:**

Seven studies met the selection criteria. Prenatal care and low birth weight were linked in four of the seven studies included in the review. The pooled odd ratio for low birth weight in the random-effects model was 0.46 (95% CI: 0.39, 0.53). The pooled odds ratio for low birth weight was 0.21 (95% CI: 0.19, 0.22) and 0.21 (95% CI: 0.19, 0.22), respectively, among pregnant women who had no antenatal care follow-up and those who had antenatal care follow up.

**Conclusion:**

Women who attended at least one antenatal care appointment were more likely than their counterparts to have a baby of normal weight. Interventions to reduce low birth weight in Africa should focus on providing adequate antenatal care and quality healthcare services to women with low socioeconomic status.

## Background

The care provided to pregnant women and adolescent girls by skilled medical professionals throughout pregnancy is known as antenatal care (ANC). Risk identification, as well as the prevention and management of diseases associated with pregnancy or other conditions that may occur concurrently, is the essential component of ANC (1). Although it is widely accepted that ANC should be tailored to each patient, there is a lack of explicit definitions of the practices required to support normal pregnancy outcomes, particularly in developing countries where resources are frequently scarce. It is due to the lack of credible studies on the effects of antenatal care continuity, availability, and substance on pregnancy outcomes like low birth weight (LBW), postnatal morbidity, and mortality (2).

The World Health Organization (WHO) defines LBW as a baby’s weight of less than 2.5 kilograms. Over 20 million births are thought to have LBW each year, accounting for 15 to 20% of all babies born worldwide. Additionally, there are regional variations in the percentage of LBW, with South Asia having a 28 percent rate, Sub-Saharan Africa having a 13 percent rate, and Latin America having a 9 percent rate (3, 4). Twenty-two million babies were born in the world in 2013. Of these, 16% of babies had LBW, with 96% of births found in developing countries (5). A study in Nepal in 2011 found that the prevalence of LBW was 15.4% (6). It was found that LBW infants were more likely to pass away in the first year. Small-for-gestational-age birth (born before 37 weeks of pregnancy), intrauterine growth retardation, or a combination of the two can result in LBW (7). Neonatal mortality and low birth weight were found to be linked, indicating a serious public health issue. A thorough review of the literature up to 2011 and a meta-analysis revealed an odds ratio of 8.5 for neonatal mortality in full-term (37 gestational weeks) newborns weighing 2.5 kg (8). As revealed by a cohort study in Brazil between 2011 and 2012, LBW was one of the factors associated with neonatal mortality (9). Furthermore, asthma and hypertension were two morbidities linked to LBW (8, 10).

Recently published research suggested that ANC attendance was related to (11, 12). The WHO recommended that ANC visits throughout pregnancy time must be at least four; however, a new model unveiled in 2016 recommends that the minimum contact must be at least eight (13). There is compelling evidence that skipping ANC visits or skipping fewer visits than recommended increased the risk of LBW by four (13). This viewpoint was supported by the fact that ANC provides a channel for delivering a variety of therapies to pregnant women that improve maternal and fetal health outcomes (14).

In impoverished nations, the highest LBW cases were caused by intrauterine development retardation, while preterm delivery was like in wealthy countries (4, 15). Aside from the negative outcomes of increased neonatal morbidity and mortality, restricted growth and cognitive development, and a higher risk of chronic illness development later in life (16). LBW has been linked to an increased risk of non-communicable diseases such as diabetes and cardiovascular disease later in life (17, 18).

Low birth weight (LBW) was associated with at least five to eight missed ANC appointments, no ANC services during the first trimester, and a lack of availability of certain ANC supplies (11, 19). Aside from that, there is disagreement about how ANC therapies influence maternal and neonatal health outcomes (20, 21). To date, ANC alone has not been proven to improve birth outcomes but other healthcare programs reduce perinatal mortality (22) and newborn morality is also reduced when study models compared with ANC visits (23).

On the other hand, insufficient and no ANC had no impact on perinatal mortality (24), while improved ANC had no effect on perinatal or neonatal mortality (25). As a result, this systematic review and meta-analysis aimed to evaluate the effectiveness of ANC in reducing the rate of low birth weight (LBW) among children in Africa.

## Methods and materials

### Search strategy

A thorough search of the literature was employed using databases (PubMed, Scopus, the Cochrane Library, and Hinari Direct) and search engines such as Google Scholar. Scholars with extensive experience in systematic reviews conducted the initial search, and GTE independently screened titles, abstracts, and full texts. In the event of a disagreement, another reviewer was asked to settle it. The initial search terms were LBW, ANC (effect, prenatal care, antenatal care, infant, low birth weight, and low-birth-weight infant, and Africa).

In the search strategy, a combination of keywords related to LBW, terms related to study design (prevalence, epidemiology, cross-sectional study, observation follow-up study), and title, title/abstract, or medical subject heading was developed. Additionally, relevant literature was identified by searching the reference lists of full-text articles and grey literature on Google.

### Eligibility criteria

We settled on the following criteria to incorporate studies in the review: (1) women who were delivering or labouring, had newborn babies, were pregnant women, or were postpartum women who had live births, (2) studies in analytical cross-sectional and observational follow-up study designs, (3) the study reported the outcomes of low birth weight, (4) the use of ANC was regarded as a factor or exposure for the outcomes, (5) the article was published in English, and (6) the study will be published? (Is it only published? (What about grey literature?)) until May 15, 2022.

### Risk of bias assessment

The selection of the articles was based on the standardized critical appraisal instrument adapted from Hoy et al.’s risk of bias tool (26). The tool has 9 items, with a maximum score of nine and a minimum of zero. The overall risk of the bias has been leveled into three categories: 0–3 = low risk, 4–6 = moderate risk and 7–9 = high risk.

### Data extraction and outcome of interest

The author extracts the data and compared the results. Discrepancies were resolved by discussion, or the six reviewers made the decision. The primary authors of the eligible studies were contacted through their email or phone for further clarification about the data. We extracted the following data from each study:

- Author(s) and years of publication
- Study design (cross-sectional and observational follow-up)
- Country of the region and participants (mothers who give birth, pregnant women, all live births, and women born 5 years prior)
- Prevalence estimates reported stratified by the weight of the child and ANC

The primary outcomes were pooled of LBW prevalence, and the secondary outcome was identifying the effect of antenatal care on LBW and investigating.

### Reliability

The second reviewer was blinded to the primary reviewer’s (GTE and DAA) decisions on article selection, data extraction, and risk of bias assessment. Any differences were solved through discussion; otherwise, another person was available to arbitrate any issues that remained unresolved.

### Analysis of the data

The relevance of each study was assessed based on its topic, objectives, and methodology. An initial descriptive analysis of the studies has been employed. Heterogeneity between estimates was assessed using the I^2^ statistic; an I^2^ value of above 75% indicates considerable heterogeneity (27–29). Potential influences on the prevalence estimate were investigated using sensitivity analyses. Where studies allowed, we descriptively compared prevalence estimates by sex, first author’s last name, publication year, study design, site, sample size, study duration, study population, outcome, and comparison groups were all recorded for each study. The exposure variable was divided into two categories: “No ANC visit at all” and “One or more ANC visits. Quantitative papers were pooled in a statistical meta-analysis using the STATA version 14. Both odd ratios and heterogeneity were considered statistically significant if the value of p was less than 0.05. Egger’s test for small-study effects (*p*-value <0.05) was used to look into possible publication bias (30).

### Outcome measurement

The WHO defines low birth weight as a weight of less than 2,500 g. But the length of the pregnancy and the rate of fetal growth affect the birth weight (31). When a newborn’s weight was less than 2,500 grams, LBW was considered (31, 32).

### Patient and public involvement

There was no patient or public involvement in this study.

## Result

### The review processes

**Figure 1:**
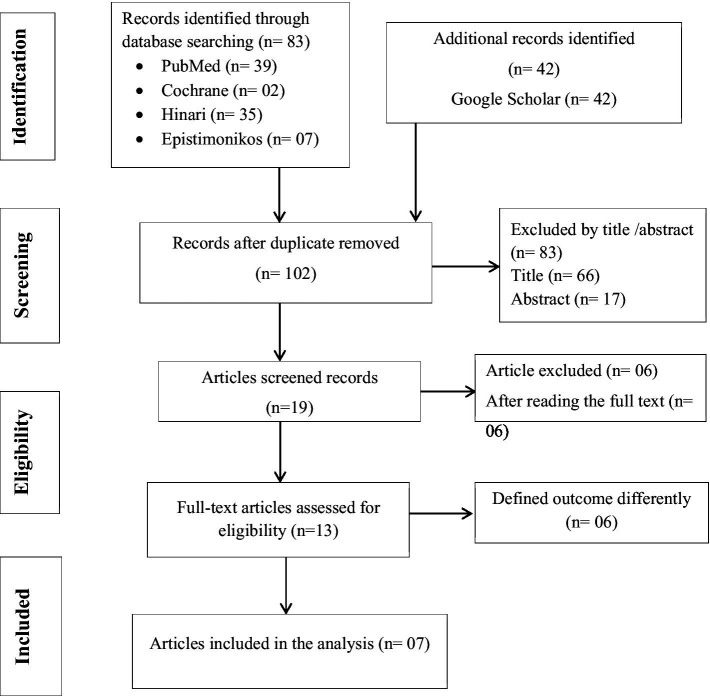
PRISMA flow chart for identifications of studies that were included in the systematic review and Meta-analysis in Africa.

The initial database search generated 125 articles. After removing duplicates by title and abstract, 83 remained. All 83 articles were considered for the full-text review. Then, after the full text of 19 articles was reviewed, 12 articles were excluded (their outcomes were not directly related to our outcomes of interest), and seven articles were included for both the systematic review and meta-analysis (Figure 1).

### Characteristics of the included studies

Table 1 lists the characteristics of the seven primary studies that were a part of this review. In diverse regions of Africa, four cross-sectional studies, one retrospective follow-up study, one case–control study, and one prospective cohort study were conducted.

**Table 1:** Characteristics of the individual studies included in this systematic review and meta-analysis, 2022.

Author	Publication year	Study design	Study area	Sample size	Response rate	Proportion	Quality status
Weyer et al.	2022	Cross-sectional	Sub-Saharan Africa	33,585	99	0.057	Low risk
Sema et al.	2019	Cross-sectional	Ethiopia	420	97.4	0.21	Low risk
Banchani et al.	2020	Cross-sectional	Ghana	25,304	99	0.077	Low risk
Shiferaw et al.	2018	Cross-sectional	Ethiopia	605	100	0.083	Low risk
Assefa et al.	2012	Observational follow-up	Ethiopia	1,295	73.8	0.283	Low risk
Olusnaya	2009	Observational follow-up	Sub-Saharan Africa	4,408	90	0.102	Low risk
Teshotetsi et al.	2019	Observational follow-up	South Africa	1,073	77	0.048	Low risk

A total of seven studies with 66,690 participants were included. About a quarter (33.3%) of the studies were from Ethiopia (33–35), one from South Africa (36), and Sub-Saharan Africa contributed two studies each (15, 37), and one was from Ghana (38). All included studies were cross-sectional and observational and published between 2009 and 2022. The sample sizes ranged from 420 in Ethiopia to 33,585 in Sub-Saharan Africa. Africa had the lowest and highest rates of LBW, 4.8 and 21.3%, respectively. Except for two studies using data from the Demographic Health Survey (DHS), all the studies were done in healthcare settings.

Of the seven included studies, five of them were institution based, while the rest were demographic and health surveys. Five studies reported the effect of ANC on low birth weight, one anthropometric measurement, and the rest general LBW (Table 1). To diagnose LBW, The weight is compared with the baby’s gestational age and recorded in the medical record. The criteria of WHO were used (birth weight less than 2,500 grams (5 pounds, 8 ounces) is diagnosed as low birth weight). Babies weighing less than 1,500 grams (3 pounds, 5 ounces) at birth are considered to have very low birth weight (31, 32).

### Meta-analysis

**Figure 2:**
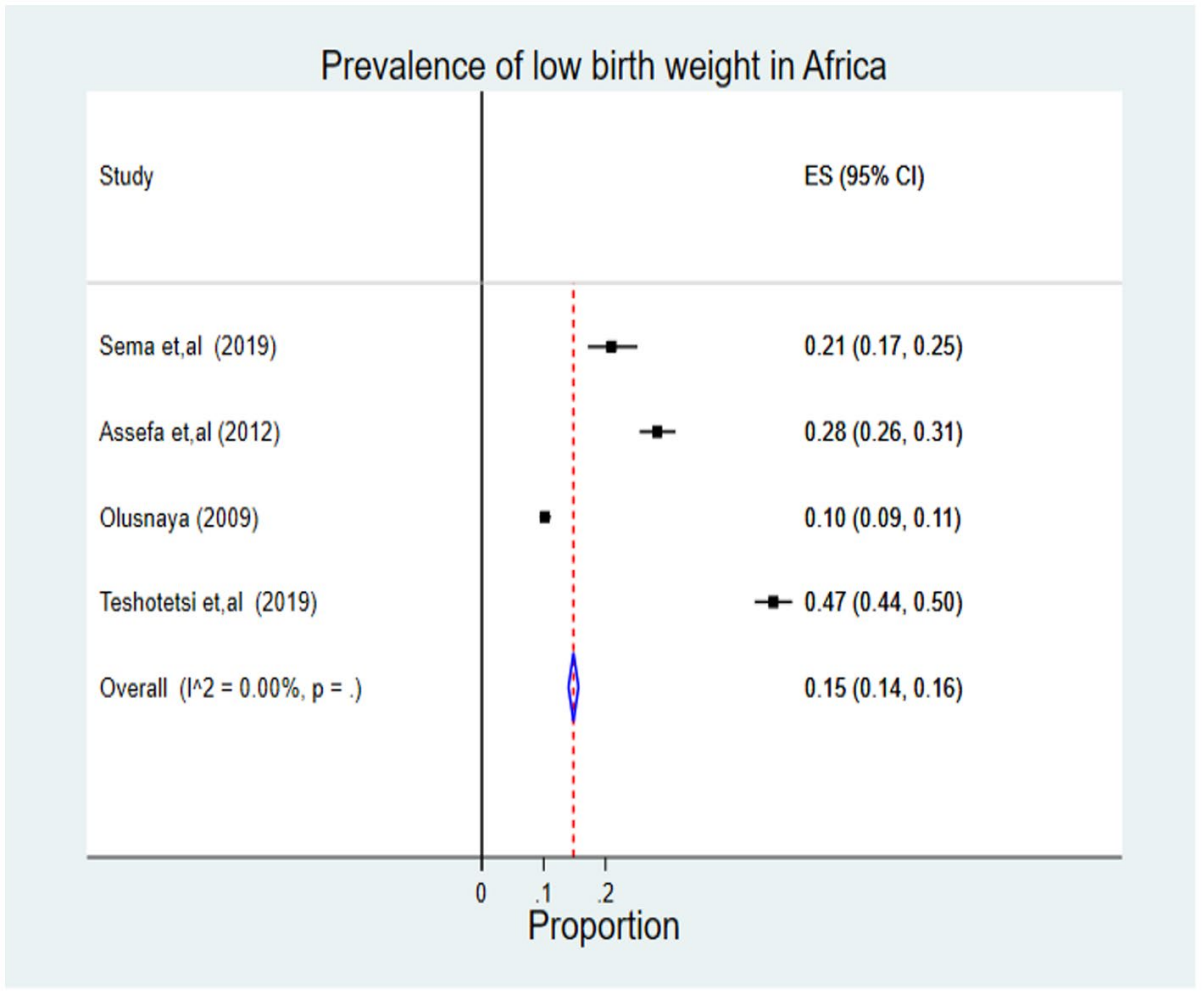
Forest plot of the pooled prevalence of LBW in Africa, 2022.

In the estimation of the pooled effect of ANC on LBW among ANC-attendant women in Africa, seven studies were used, and a total of 66,690 pregnant women and mothers who give birth were participants. The forest plot results of seven included studies showed that the overall pooled prevalence of LBW in Africa was 15% (95% CI: 0.14, 0.16). There is no heterogeneity, as evidenced by the I-squared (I^2^) statistics (I^ʌ^2 = 0.00%, *p* = .). Hence, a fixed-effect model was used to estimate the overall pooled effect size of ANC on LBW among ANC-attendant women in Africa (Figure 2).

**Figure 3:**
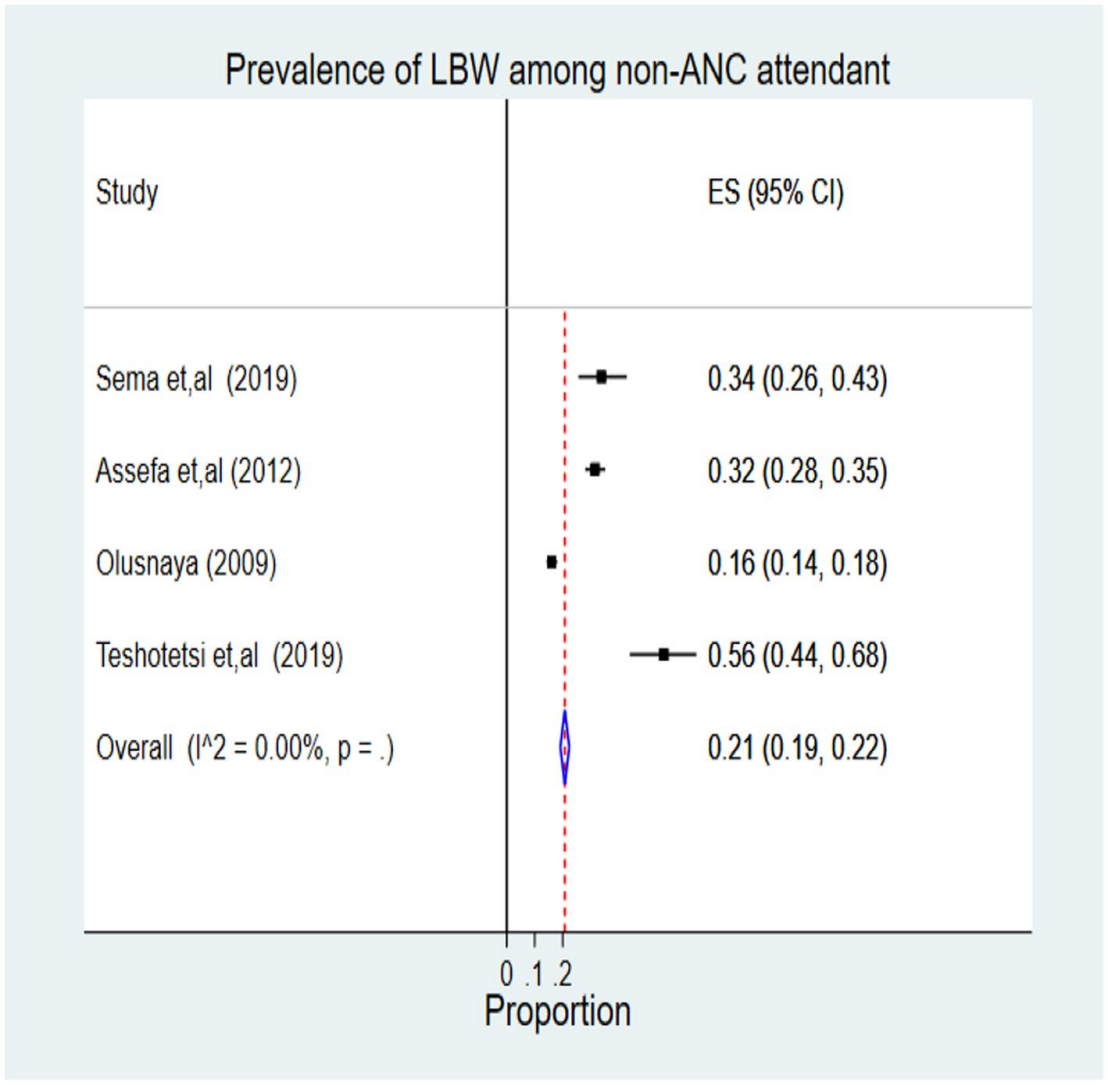
Forest plot of pooled prevalence of LBW among non-ANC attendant women in Africa, 2022.

From this review, among non-ANC attendant women, 533 cases of LBW were identified among 2,348 newborn children, representing 21% (95% CI: 0.19, 0.22). According to the I^2^ statistics, heterogeneity is not an issue (I^ʌ^2 = 0.00%, *p* = 0.01) (Figure 3).

**Figure 4:**
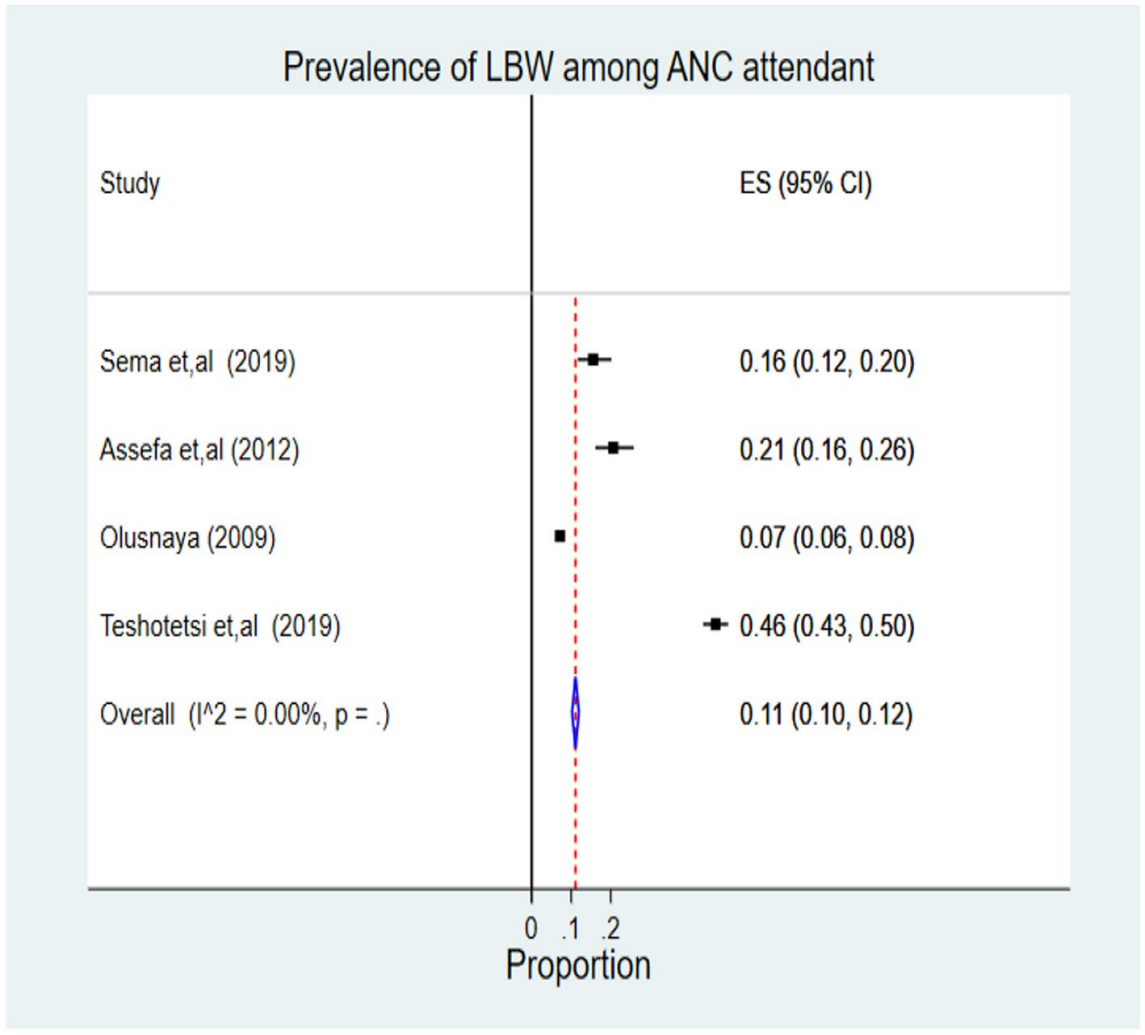
Forest plot of pooled prevalence of LBW among ANC attendant women in Africa, 2022.

Four thousand four hundred nine newborns had 769 cases of LBW among ANC attendant mothers, which represents 11% (95% CI 0.10, 0.12). According to the I^2^ statistics, there was no heterogeneity problem (I^ʌ^2 = 0.00%, *p* = 0.01) (Figure 4).

### Subgroup analysis by study design

**Figure 5:**
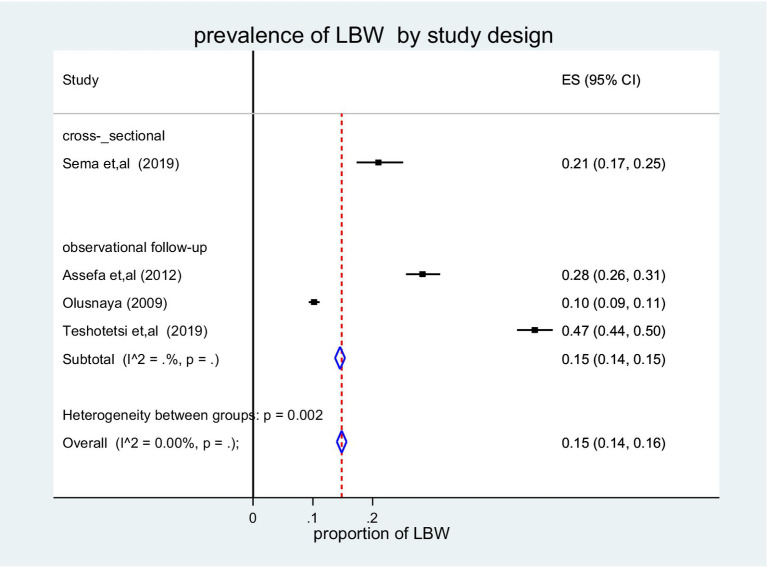
Forest plot of the pooled prevalence of LBW among children in Africa by study design, 2022.

A subgroup analysis was performed to determine the pooled prevalence of LBW in Africa based on the study design. The cross-sectional study had the greatest prevalence rate, at 21% (95% CI: 0.17, 0.25), and Figure 5 shows the I-squared statistics (I^ʌ^2 = 0.00%, *p* = .).

### The pooled effect size of ANC on low birth weight

**Figure 6:**
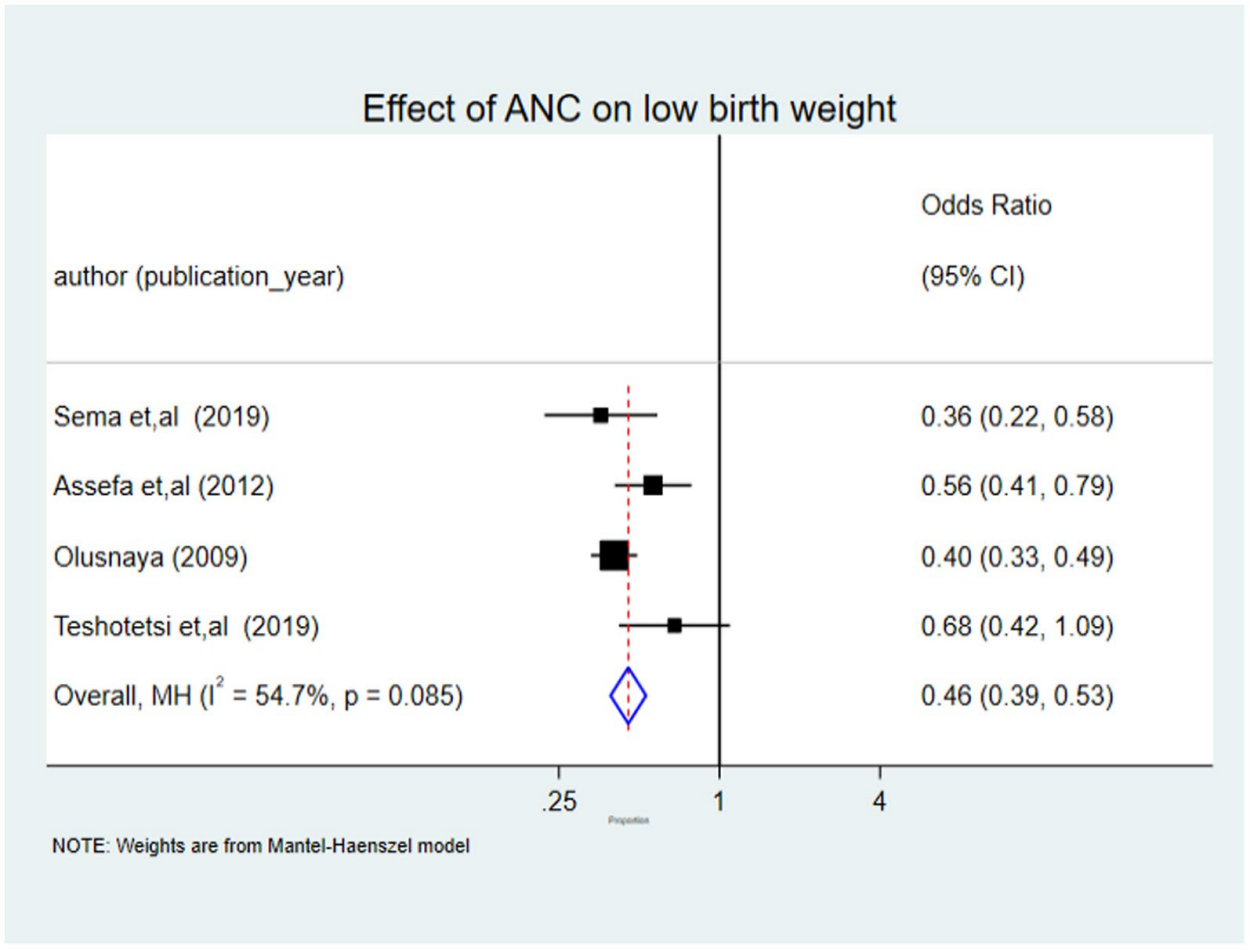
Forest plot of the pooled effect size ANC on LBW among children in Africa, 2022.

Four of the seven studies included in the analysis, each with at least one ANC visit, revealed statistically significant associations with LBW. There were 6,757 children involved in all four studies. Compared to neonates whose mothers received no ANC follow-up at all, the Mantel–Haenszel method by fixed effect model for children born to women who received at least one ANC follow-up was 0.46 (95% CI: 0.39, 0.53) with I-squared statistics of 54.7% (I^ʌ^2 = 54.7%, *p* = 0.000). According to this finding, using ANC services decrease the burden of LBW by 54% when compared to non-ANC attendants (Figure 6).

### Small study effect

Utilizing a funnel plot and Egger test, it was determined whether a potential small study effect existed. The value of *p* for egger’s test was checked (*p* = 0.131), and there was no indication of publication bias since this funnel plot demonstrated an asymmetric distribution (Table 2).

**Table 2:** Egger test of the effects size of ANC on LBW among children in Africa, 2022.

Number of studies =7				Rot MSE = 6.285		
Std_Eff	Coef.	Std.err.	*t*	*p* > |t|	[95% CI]	
slope	0487188	0.019329	2.52	0.053	−0.0009681	0.0984056
bias	6.063516	3.357926	1.81	0.131	−2.568308	14.69534

Taste of H0: no small-study effects, *p* = 0.131.

## Discussion

The purpose of this study was to determine the efficacy of targeted ANC in reducing LBW in newborns from African populations. Seven major studies testing ANC with a variety of groups, including pregnant women, labouring women, and postpartum moms, as well as their newborn children’s weight, were found to be eligible. The benefits of ANC delivered by skilled attendants for the health of babies have been documented throughout Africa. In LMICs, a variety of tactics and strategies have been used to improve the effectiveness of ANC. Most LMICs, including Africa, have adopted WHO’s focused ANC approach, which was created in the 1990s (39). In this meta-analysis, a total of 66,690 children were included to estimate the pooled effect size of ANC on low birth weight. The seven studies were conducted in different countries on the continent. All studies were conducted in a health institution setting, and among a total of 66,690 children, 533 and 769 low birth weight newborns were identified among non-ANC attendant and ANC attendant women, respectively. The risk of bias assessment was done for both cross-sectional and observational follow-up studies independently using JBI risk of bias assessment tools, and all studies had a low risk of bias.

The association between low birth weight and prenatal care utilization is clinically plausible and has been noted previously. A child’s birth weight is an important indicator of the child’s vulnerability to the risk of childhood illnesses and the chances of survival. Although, LBW is one of the main risk factors for infant morbidity and mortality. In this study, the pooled prevalence of LBW in Africa was 15%. This finding is higher than the study conducted in Brazil (40) but lower than the other study conducted in the United States (18.9%) (41), Bangladesh (18%) (42), Spain (17%) (43), India (25%) (44) and developing countries (19%) (43) (Countries where it is an important public health problem). The possible explanation for this disparity may be due to the existence of healthcare infrastructure disparities and different levels of healthcare provision around the world. Another possible explanation may be the delayed initiation of prenatal care due to not receiving health services, maternal age, parity, or socioeconomic status. Poverty affects one’s ability to access medical care, travel for referrals, and purchase food and sanitary products, all of which have an indirect effect on a baby’s birth weight (45, 46).

Different factors affect LBW among pregnant mothers. Risk factors for low birth weight include, for example, the location of the kitchen in the living room, an iron intake of less than 180 tablets, a weight gain of less than 6.53 kg during the second and third trimesters, comorbidity during pregnancy, attendance at ANC, and preterm birth (47, 48). Attendance of at least one ANC follow-up by pregnant women had a statistically significant effect on LBW. In this study, women who had at least one ANC visit with a qualified attendant in Africa had a 54 percent lower risk of LBW. The study’s main finding was that even limited ANC (as little as one visit) results in better newborn weight than no ANC, and encouraging pregnant women to seek ANC would have a significant impact on the LBW rate and would be an essential strategy to include in planning initiatives aimed at reducing LBW. This result is consistent with studies from other countries (49, 50), and other population-based birth registry results from the global network in Africa, India, Pakistan, and Guatemala (51). A study in Asia found that women who had ANC visits and delivered in a health institution had a lower risk of LBW (52).

Low birth weight (LBW) is related to the quality and sufficiency of prenatal care (ANC). Additionally, it is a result of intrauterine growth restriction or premature birth. Pregnant women receive a variety of services during ANC visits, including tetanus toxoid-diphtheria immunization and intermittent preventative therapy in pregnancy (IPTp), both of which are crucial for the health of the expectant mother and the unborn child (5, 15). A subgroup analysis and a thorough database search were both performed to determine whether any particular study level factor best described the results. The vast sample size of the analysis allowed it to identify the impact of ANC on LBW in Africa because the evaluation covered all research done in Africa. They were done in English and were cross-sectional and observational studies with inherent biases, the systematic review and meta-analysis were constrained. Articles based on the number of visits were excluded because they did not identify zero visits, making it impossible to pool the overall effect size of ANC for those studies.

### Limitations of the study

The evaluation of this study covered all research done in Africa, and the large sample size of the analysis allowed it to identify the impact of ANC on LBW in Africa. However, the studies were cross-sectional and observational with inherent biases, and the systematic review and meta-analysis were limited. Articles based on the number of visits were excluded because they did not identify zero visits, making it impossible to pool the overall effect size of ANC for those studies.

## Conclusion

The frequency and follow-up of prenatal care visits demonstrated a positive correlation with birth weight. Encouraging pregnant women to seek prenatal care, even when they are in their third trimester, can be beneficial. Furthermore, it is warranted to point out the necessity of enhancing communication, education, and information activities. A well-designed prospective follow-up study should be conducted to determine whether a minimal number of visits are required to increase birth weight.

## Data availability statement

The original contributions presented in the study are included in the article/supplementary material, further inquiries can be directed to the corresponding author.

## Author contributions

GTE and DAA were responsible for the inception of the idea and study. The authors participated from the inception of the study to the final manuscript’s writing, reviewing, and editing. AHT, MF, AN, AY, WA, and MTE were also involved in the article selection, statistical analysis, and writing and editing of the manuscript. All authors contributed to the article and approved the submitted version.

## Conflict of interest

The authors declare that the research was conducted in the absence of any commercial or financial relationships that could be construed as a potential conflict of interest.

## Publisher’s note

All claims expressed in this article are solely those of the authors and do not necessarily represent those of their affiliated organizations, or those of the publisher, the editors and the reviewers. Any product that may be evaluated in this article, or claim that may be made by its manufacturer, is not guaranteed or endorsed by the publisher.

## Abbreviations

ANC: Antenatal Care
CI: Confidence Interval
DHS: Demographic Health Survey
IUGR: Intrauterine Growth Restriction
JBI: Joanna Briggs Institute
LBW: Low Birth Weight
LMICS: Low-and-Middle-Income Countries
PICO: Population, Intervention, Comparison, Outcome
PRISMA: Preferred Reporting Items for Systematic Review and Meta-analysis statement
SSA: Sub-Saharan Africa
WHO: World Health Organization
